# Sestrin2: Its Potential Role and Regulatory Mechanism in Host Immune Response in Diseases

**DOI:** 10.3389/fimmu.2019.02797

**Published:** 2019-12-04

**Authors:** Li-Xue Wang, Xiao-Mei Zhu, Yong-Ming Yao

**Affiliations:** ^1^Trauma Research Center, Fourth Medical Center of the Chinese PLA General Hospital, Beijing, China; ^2^State Key Laboratory of Kidney Disease, The Chinese PLA General Hospital, Beijing, China

**Keywords:** Sestrin2, immune response, autophagy, endoplasmic reticulum stress, immune cell

## Abstract

Sestrin2 (SESN2), a highly evolutionarily conserved protein, is critically involved in cellular responses to various stresses. SESN2 has a protective effect on physiological and pathological states mainly via regulating oxidative stress, endoplasmic reticulum stress, autophagy, metabolism, and inflammation. In recent years, breakthrough investigations with regard to the regulation and signaling mechanisms of SESN2 have markedly deepened our understanding of its potential role as well as its significance in host response. However, the functions of SESN2 in the immune system and inflammation remain elusive. It has been documented that many immune cells positively express SESN2 and, in turn, that SESN2 might modulate cellular activities. This review incorporates recent progress and aims to provide novel insight into the protective role and regulatory pathway of SESN2, which acts as a potential biomarker and therapeutic target in the context of various diseases.

## Introduction and Background

Sestrins (SESNs), a highly evolutionarily conserved protein family, can be induced by various stresses, including oxidative stress, DNA damage, hypoxia, and nutritional stress (1–5). The SESN proteins play important roles in protecting organisms and cellular homeostasis from stress injury mainly by downregulation of reactive oxygen species (ROS) accumulation and the mammalian target of rapamycin protein kinase (mTOR) signaling (5–9). Increasing numbers of studies have documented that three SESN genes, *SESN1* (PA26), *SESN2* (Hi95), and *SESN3*, have been identified in most vertebrates, while only one SESN gene has been found in invertebrates, and no SESN genes have been found in yeast (6). The first gene of this family, named *SESN1* (PA26), was initially discovered in 1999 as a p53 transcription factor in the cellular response to genotoxic stress from treatment with ultraviolet (UV), γ-irradiation, and cytotoxic drugs in 1999 (2). Several years later, SESN2, which is highly homologous to the *SESN1* gene, was identified under hypoxic conditions, while p53 was dispensable to this process (3). *SESN3* was observed as a gene that was activated by the forkhead transcription factor (FoxO) under conditions of energy crisis (9, 10). Although these proteins share high similarity of biological structure with each other and show some common effects in modulation of 5′-adenosine monophosphate-activated protein kinase (AMPK) and mTOR, there are also many differences. Additionally, they lack an obvious structural effector domain, and no clear function has been found during the years.

**Figure 1:**
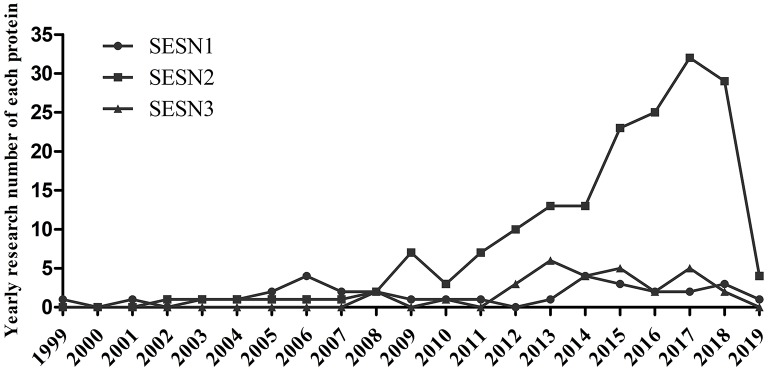
Current research situation of the SESN family. PubMed search in May 2019.

Growing evidence has demonstrated that the three members of the SESNs perform diverse functions (Table 1). SESN1 is associated with autophagy-related genes and can inhibit mTORC1 or ROS in cells (5). SESN2 has an antioxidant function, activates AMPK, and inhibits mTORC1 signaling (5, 6). SESN3 suppresses mTORC1 activity and maintains Akt activity by activating the AMPK/TSC1/2 axis (10, 49). Among these members, SESN2 has been the most profoundly investigated since its discovery in 2002; investigations concerning the structure or function of SESN1 and SESN3 have been limited. Previous studies have revealed that the structure of human SESN2 (hSESN2) displays three subdomains, namely, SESN-A, SESN-B, and SESN-C (50–52). The domain of SESN-A functions as an active alkyl hydroperoxide reductase, which is critical for parts of its antioxidant effect. The site of SESN-B, a leucine-binding site, illuminates the way that SESN2 interacts with leucine. SESN-C interacts with GTPase-activating protein complex for Rag (GATOR2) and performs a pivotal role in the regulation of AMPK and mTORC1 signaling by hSESN2. Current research on the SESN family is shown in Figure 1.

**Table 1:** Identified functions of three member of the SESN family.

**System diseases**	**SESNs**	**Type of disease**	**Effect**	**Mechanisms**	**References**
Immune system	SESN1, SESN2, SESN3	T-cell senescence	Pro-aging function in T lymphocytes	Activating Erk-JNK-p38 MAPK complex	(11)
	SESN2	Sepsis and sepsis shock	Protect the host from sepsis	Inducing mitophagy and inhibiting prolonged inflammasome activation	(12)
Liver disease	SESN2	Liver damage, hepatosteatosis	Hepatoprotective effect	Inhibiting oxidative stress response and prolonged ER stress	(13–18)
	SESN3	Nonalcoholic steatohepatitis	Protect against diet-induced non-alcoholic steatohepatitis	Suppressing of TGF-β-Smad3 signaling	(19)
Ischemia–reperfusion (I/R) injury	SESN2	Cardiovascular/ renal/cerebral I/R injury	Protective capabilities against I/R injury	Promoting AMPK activation or activating autophagy and mitophagy	(20–25)
Neurodegenerative disease	SESN2	Neuropathic pain, Alzheimer's disease, Parkinson's disease, Huntington' disease	Neuroprotection	Antioxidant against ROS, activating AMPK-dependent autophagy and suppressing mTORC1	(26–29)
	SESN3	Temporal lobe epilepsy	Modulating brain inflammation and epilepsy	Regulating pro-convulsant gene network	(30)
Cardiovascular disease	SESN1	Cardiac hypertrophy	Anti-hypertrophic function	Activating autophagy via regulating AMPK/mTORC1	(31)
	SESN2	Cardiac hypertrophy, myocardial infarct, ischemic cardiomyopathy and dilated cardiomyopathy, atherosclerosis	Protective effect on cardiovascular remodeling and regeneration	Inhibiting ERK1/2 signaling, enhancing antioxidant effect on the Nrf2/Keap1 pathway	(32–35)
Lung disease	SESN2	COPD, emphysema	Negative effect on pulmonary function	Regulating the TGF-β and mTOR pathways	(36, 37)
Cancer	SESN1	Thyroid cancer, follicular lymphoma	Tumor inhibitory effect	Regulating the p53-AMPK-mTOR signaling pathway	(38, 39)
	SESN2	Most cancers in diverse organ systems	Tumor inhibitory effect	Activating MAPK8/JNK1, inhibiting the mTORC1 pathway	(40–44)
	SESN3	Chronic myeloid leukemia, lung cancer	Anti-leukemic responses, tumor inhibitory effect	Inhibiting the PI3K/AKT/mTOR pathway	(45, 46)
Aging	SESN1	Delay aging and age-associated disorders	Regulating life span	Suppression of ROS accumulation	(47)
	SESN2 SESN3	Insulin resistance	Control of lipid and glucose metabolism and liver insulin resistance	Inhibiting mTORC1-S6K signaling	(48)

**Figure 2:**
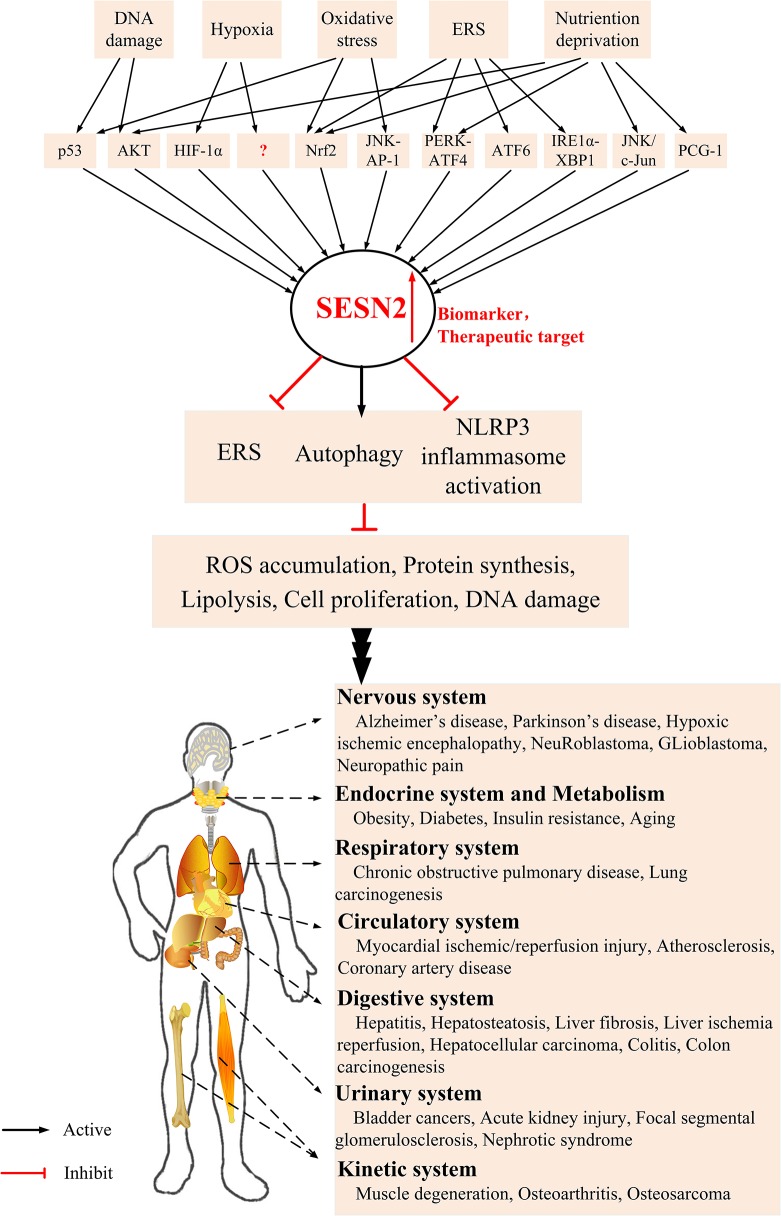
The underlying mechanism of SESN2 induction and potential role of SESN2 as a therapeutic target in diverse systems. Various stress insults increase the expression of SESN2 via regulation of several critical transcription factors. Upregulation of SESN2 inhibits ROS accumulation, protein synthesis, lipogenesis, cell proliferation, and DNA damage through alleviating the extent of ERS, activating autophagy, or relieving inflammasome activation. SESN2, with several modulatory effects, acts as an advantageous therapeutic target and provides protective roles in various inflammatory disorders.

SESN2 was initially observed as a downstream effector of p53, and a myriad of adverse environmental stresses can induce SESN2 expression, such as oxidative stress, endoplasmic reticulum (ER) stress (ERS), energetic stress, and age- and obesity-associated metabolic pathologies (9, 53–58). It has been reported that SESN2 has pleiotropic biologic functions in cell homeostasis and metabolic homeostasis during diverse conditions by regulating autophagy, ERS, and inflammasome activity. However, the effect of SESN2 on the inflammatory and immune systems is still not yet well understood. The current review focuses on functions, pathophysiological effects, and regulatory mechanisms of SESN2 in the development of inflammatory-related diseases (Figure 2).

## Regulation of SESN2 Induction

As a stress-induced protein, SESN2 can be regulated to adapt to various metabolic stimuli at the cellular or organism level. Thus, it is pivotal to investigate how SESN2 expression is regulated during diverse physiological and pathological conditions.

### DNA Damage

DNA damage can be provoked by different stresses, and it might result in metabolic homeostasis and tissue inflammation. p53 plays a key role in the regulation of cellular responses to DNA damage, and it is involved in the modulation of cell cycle arrest, cellular apoptosis, and metabolic dysfunction (59). It was reported that SESN2 was upregulated in a p53-dependent manner in response to DNA damage and played an important role in maintaining cellular activity (3, 5). In accordance with previous studies in fibroblasts and keratinocytes, Mlitz et al. (60) found that ultraviolet-B (UVB) irradiation obviously induced DNA damage, which increased the SESN2 level, and this upregulatory response could be abolished by silencing p53. Moreover, the p53 and Akt pathways are vital for UVB irradiation-mediated SESN2 upregulation in melanocytes (61). In sebaceous gland carcinoma, p53 appeared to be required for SESN2 induction, and a low level of SESN2 was related to advanced tumor stage and poor prognosis (62).

### Oxidative Stress

Oxidative stress occurs when ROS are excessively produced and antioxidant systems are unable to balance the response, which can trigger various inflammatory mediators in inflammation-associated chronic diseases (63). Nuclear factor erythroid 2-related factor 2 (Nrf2) acts as a crucial transcription factor that can modulate antioxidant gene expression by interacting with the antioxidant response elements (AREs). Under physiological conditions, Nrf2 binds to its negative repressor Kelch-like ECH-associated protein 1 (Keap1) and is inactivated (64). SESN2, with antioxidant property, is transcriptionally induced by oxidative stress, and its upregulation is vital in attenuating oxidative damage and maintaining cellular viability and cellular functions (13, 53, 65–67).

For instance, in hepatocytes, Shin et al. (53) demonstrated that Nrf2 activators specifically upregulated the expression of SESN2 but not SESN1 and SESN3 in a time- and dose-dependent manner and that Nrf2–ARE pathway activation seemed to be essential for SESN2 induction. In turn, SESN2 might act as a positive modulator of Nrf2 signaling, which shows a beneficial effect in Nrf2-mediated oxidative stress. Interestingly, it has been reported that SESN2 is closely related to both Keap1 and the autophagy adapter p62/sequestosome-1 (65). Bae et al. (65) revealed that SESN2 protected against oxidative damage by enhancing Keap1 degradation mechanistically via p62-mediated autophagic degradation and consequent Nrf2 activation. More recently, in brown adipose tissue (BAT) metabolism, SESN2 played an antioxidant defense role in mitochondrial and BAT metabolism by inhibiting Ucp1 expression associated with ROS-mediated p38 mitogen-activated protein kinase (MAPK) activation (66, 68). In summary, these data suggest that oxidative stress can induce SESN2 expression and that SESN2 acts as a defense regulator against excessive oxidative stress to protect cells through various transcription pathways.

### Hypoxia

Hypoxia, one of the most severe metabolic insults, has lower oxygen content and pressure in cells and can lead to pathological tissue damage or inflammation. *SESN2* was originally identified as a novel gene under hypoxic conditions in a p53-independent and hypoxia-induced factor-1α (HIF-1α)-independent manner in various cells both *in vivo* and *in vitro* (3), which appears to be in contrast to other studies in which the induction of SESN2 is HIF-1α dependent (69–71). Essler et al. (70) found that hypoxia and nitric oxide (NO) strongly induced SESN2 expression in a HIF-1α-dependent manner in RAW264.7 cells and that the activation of SESN2 prevented peroxiredoxin overoxidation to protect cells. In addition, in hypoxia-ischemic encephalopathy models, severe hypoxia-ischemic injury upregulated SESN2 expression in a HIF-1α-dependent manner, and SESN2 inhibited vascular endothelial growth factor formation and attenuated brain infarction or edema (71).

SESN2 could suppress HIF-1α accumulation and hypoxia response element (HRE)-dependent gene transcription by regulating AMPK-prolyl hydroxylase (PHD) in colorectal cancer cells, showing its antitumor effect (72). A recent study reported that the upregulation of SESN2 not only by hypoxia itself but also by prolonged hypoxia caused energy stress by diverse modulators (56). Thus, hypoxia can induce the expression of SESN2, while the precise mechanisms under hypoxia remain unclear and need further studies.

### Nutrient Starvation

AMPK and mTORC1 are crucial nutrient sensors that modulate metabolic energy homeostasis at the whole-body level. When responding to stresses, SESN2 exerts a protective effect by activating AMPK and inhibiting mTORC1 signaling (5, 6). It has been documented that SESN2 is the only SESN family member that is increased under energy deficiency and that the phosphoinositide-3 kinase/Akt (PI3K/Akt) pathway, but not p53, is required for SESN2 induction (56). Meanwhile, SESN2 markedly protected against energy deprivation-induced cell apoptosis via inhibiting the mTOR pathway (56). Glucose deprivation increased SESN2 expression, which was dependent on Nrf2–ARE activation in hepatocytes, which was related to glucose deprivation-induced ROS accumulation. Thus, SESN2 might play a pivotal role in the cellular adaptive response by increasing AMPK activity to maintain mitochondrial homeostasis (73). In agreement with this study, Ding et al. (54) reported that SESN2 was the major responder that was activated among SESN family members during energy stress, and its induction mechanism was dependent on ERS transcription factors including activating transcription factor (ATF) 4 and Nrf2 but not p53. Additionally, as is known, leucine, a proteogenic amino acid, facilitates mTORC1 by the Rag GTPases, as well as their regulators GATOR1 and GATOR2. SESN2 binds to GATOR2 in cells in an amino-acid-sensitive manner (8, 74, 75). Upon amino acid depletion, the expression of SESN2 was increased, and the structure was altered, which manifested as SESN2 becoming more highly phosphorylated and the SESN2–GATOR2 interaction being much effective, leading to a suppression of the mTORC1 signaling and then regulating protein synthesis as well as autophagy (50, 75).

In cancer cells, c-Jun N-terminal kinase (JNK) pathway activation and its downstream factor c-Jun phosphorylation are required for the regulation of SESN2 transcription under serum deprivation (76). In a recent study, SESN2 expression was found to depend on peroxisome proliferator-activated receptor–coactivator (PGC)-1α activation under glucose scarcity in liver cancer cells (77).

## Regulatory Mechanisms of SESN2

In response to various stimuli, SESN2 is known to inhibit mTORC1 by activating AMPK signaling and prevent ROS accumulation in cells. Increasing evidence shows that different cell signaling pathways are regulated by SESN2, including autophagy, ERS, and inflammasomes, in response to stimuli to exert cytoprotective effects.

### Autophagy

Autophagy, a self-degradation process, is a basic metabolic mechanism to maintain cellular homeostasis and survival by degrading intracellular proteins as well as dysfunctional organelles during stresses. Increasing evidence shows that autophagy malfunction is associated with many human diseases, such as cancer, neurodegenerative diseases, liver diseases, and inflammatory diseases (78–80). As we know, pathogen-associated molecular patterns (PAMPs) may activate autophagy via regulation of mTORC1 and AMPK. Based on the critical role of mTOR in the regulation of autophagy, it is our belief that SESN2 is closely related to autophagy. Many studies have revealed that SESN2 can regulate autophagy by modulating the mTORC1/AMPK pathway in various cells (81, 82).

For example, SESN2, a novel positive modulator of autophagy, was increased in p53-sufficient cells but not in p53-deficient cells when treated by different autophagy inductors, and knockdown of SESN2 decreased the induction of autophagy (83). Hou et al. (84) reported that in a rotenone-induced Parkinson's disease model, the mRNA and protein levels of SESN2 were significantly upregulated in a p53-dependent manner and that silencing of SESN2 suppressed autophagy while increasing the accumulation of autophagy substrates, which manifested as downregulation of light chain 3II (LC3II), exacerbation of p62 accumulation, and inhibition of AMPK phosphorylation. In contrast, overexpression of SESN2 could enhance autophagy activity, as evidenced by enhanced phosphorylation of AMPK and p62 or α-synuclein degradation, thereby protecting cells from rotenone cytotoxicity insults. These results indicate that SESN2 is involved in the regulation of autophagy, which might be closely associated with AMPK phosphorylation. Although it has been reported that SESN2 could regulate mTOR via the AMPK–TSC2 pathway and Rag GTPase, the mechanisms by which SESN2 modulates autophagy activity remain to be elucidated.

Recently, Ro et al. (85) reported that Unc-51-like protein kinase 1 (ULK1) and p62 were new binding partners for SESN2 and that SESN2 was physically associated with the C-terminal domain of ULK1 to provoke its function. Moreover, SESN2 could interact with the ULK1–Atg13–FIP200 complex to promote p62 phosphorylation, which contributed to eliminating malfunctioning mitochondria and ubiquitinated proteins and modulating the Keap1–Nrf2 pathway. Therefore, the induction of autophagy can markedly enhance SESN2 expression, and SESN2 interacts with multiple autophagy-related proteins to modulate autophagy activity.

### ER Stress

The ER is highly sensitive to alterations and disturbances in ER homeostasis associated with human diseases. The unfolded protein response (UPR), triggered by the accumulation of unfolded or misfolded proteins within the lumen of ER in various stresses, determines the cell destiny by adjusting the balance between cell adaption to stress and cell death (86, 87). The UPR has evolved at least three branches involving PER-like ER kinase (PERK)-eukaryotic translation initiation factor 2α (eIF2α), inositol-requiring enzyme 1 (IRE1)-X-box binding protein 1 (XBP1), and ATF6, and all three pathways can be triggered by different stresses to restore protein-folding homeostasis and regulate ER homeostasis (88).

Currently, increasing data have demonstrated that SESN2 can be upregulated under the ERS response and has a protective effect on ERS-associated diseases (14, 15, 32, 89). For instance, Bruning et al. (89) found that in cancer cells, ERS-upregulated SESN2 was associated with the transcription factor ATF4 and that in return SESN2 inhibited the main regulatory components of the mTOR complex to regulate autophagy homeostasis. Under glucose deprivation, SESN2 was augmented through ERS induction and mechanistically via the transcription factors ATF4 and Nrf2 activation but not via p53. SESN2 protected cell viability and cell death against glucose deprivation through regulation of cellular energy metabolism and mitochondrial homeostasis (54). Similarly, in a methionine/choline-deficient diet-induced hepatic steatosis model, SESN2 expression could be enhanced in a manner that was dependent on the PERK/eIF2α/ATF4 pathway, and it ameliorated hepatic steatosis progression through autophagy activation (16). In agreement with these findings, a study by Saveljeva et al. (55) revealed that the PERK/eIF2α/ATF4 and IRE1/XBP1 pathways of the UPR were responsible for ERS-induced SESN2 expression in a p53-independent manner, and knockdown of perk or xbp1 in MCF7 and HCC1806 cells reduced SESN2 expression. In addition, SESN2 deficiency significantly exacerbated persistent protein synthesis and the extent of ERS, inhibited ERS-induced autophagy, and augmented ERS-related cell death.

**Figure 3:**
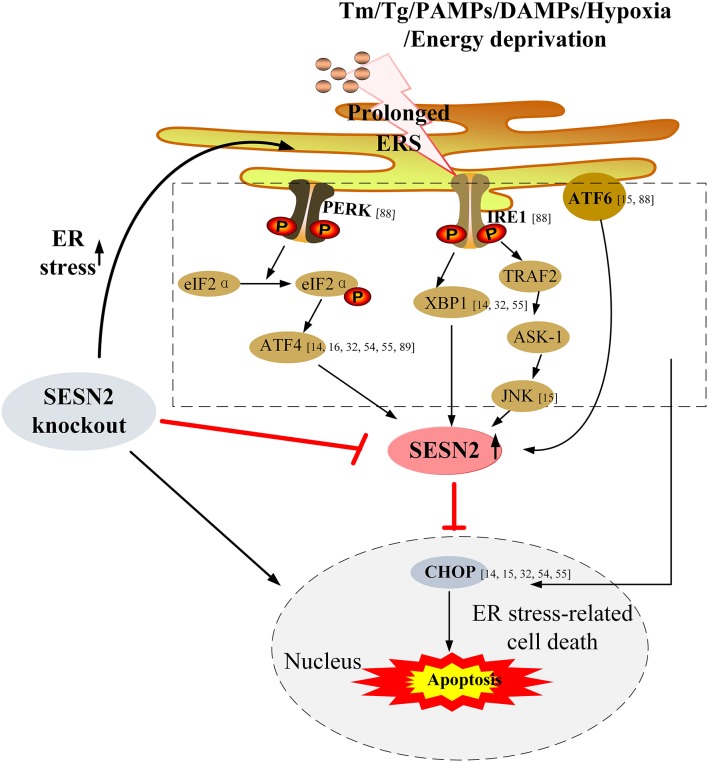
Upregulation of SESN2 under ERS and its protective effect on ERS-associated cell death. Under conditions of stress, ERS is activated due to accumulation of unfolded or misfolded proteins within ER lumen, and then the three branches of the ERS pathway involving PERK (via eIF2α and ATF4), IRE1 (through XBP1, TRAF2, and JNK), and ATF6 upregulate SESN2 expression. SESN2 exerts homeostatic feedback to attenuate the ERS response or ERS-induced cell death. Overexpression of SESN2 reduces ERS-related cell death, while knockout of SESN2 exacerbates the extent of ERS, which results in enhanced ERS-mediated cell apoptosis. Tm, tunicamycin; Tg, thapsigargin; PAMPs, pathogen-associated molecular patterns; DAMPs, damage-associated molecular patterns; PERK, PER-like endoplasmic reticulum kinase; eIF2α, eukaryotic translation initiation factor 2α; ATF4, activating transcription factor 4; IRE1, inositol-requiring enzyme 1; XBP1, X-box binding protein 1; TRAF2, TNF-α receptor-associated factor 2; ASK-1, apoptosis signal-regulating kinase 1; JNK, c-Jun N-terminal kinase; ATF6, activating transcription factor 6; CHOP, C/EBP homologous protein.

Furthermore, Jegal et al. (15) elucidated that ERS-induced SESN2 expression was dependent on the ATF6 transcription factor in hepatocytes and that in turn overexpression of SESN2 alleviated ERS-mediated cytotoxicity and attenuated liver damage by downregulation of phosphorylation of JNK and p38 MAPK and cleavage of PARP both *in vitro* and *in vivo*. Taken together, these transduction pathways are involved in the enhancement of SESN2 during ERS, and SESN2 might serve as an important regulator that exerts homeostatic feedback to attenuate the ERS response or ERS-induced cell death. These studies highlight the pivotal pro-survival role of SESN2 during cell stress. The signaling pathways that interconnect the regulation between SESN2 and ERS are shown in Figure 3.

### Inflammasomes

Jurg Tschopp's group first presented the concept of inflammasomes in 2002, and inflammasomes are complexes of multiple proteins that can activate caspase-1 and cause maturation of the substrates IL-1β and IL-18 (90). It is well-known that IL-1β and IL-18 act on various immune cells to regulate the immune response. The hyperactivation of the inflammasome ultimately leads to inflammation, cell death, and tissue injury. NOD-like receptor (NLR) family, pyrin domain containing 3 (NLRP3) is one of the most comprehensively characterized inflammasome proteins, and its prolonged activation plays an important role in the pathogenesis of diverse inflammatory diseases (91).

It has been reported that SESN2 suppressed the excessive activation of the NLRP3 inflammasome and mitochondrial damage, and silencing of SESN2 exacerbated NLRP3-dependent caspase-1 activation and the secretion of IL-1β and IL-18 after treatment with lipopolysaccharide (LPS). The inhibitory effect of SESN2 on the inflammasome was related to the maintenance of mitochondrial homeostasis by autophagosome formation and mitophagy activation. Mechanistically, SESN2 promoted perinuclear clustering-damaged mitochondria via mediating the aggregation of SQSTM1 and its binding to Lys63-linked ubiquitins on the damaged mitochondrial surface. SESN2 expression could increase ULK1 protein levels and contribute to enhanced mitophagic activity (12).

## SESN2 and Immune Cells

**Figure 4:**
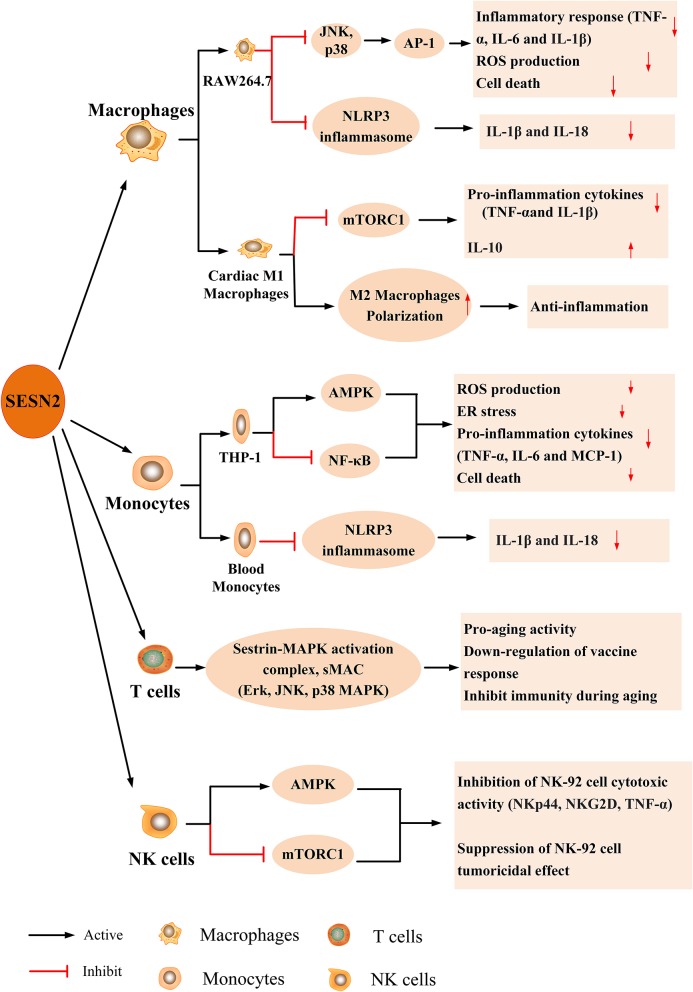
The expression and effect of SESN2 on immune cells. SESN2 has been found to be expressed in multiple immune cells such as monocytes, macrophages, NK cells, and T lymphocytes. The expression of SESN2 exerts a beneficial effect on the function of immune cells through activating AMPK, inhibiting mTORC1 signaling, suppressing JNK pathway activation, or alleviating the prolonged activation of NLRP3 inflammasome.

The possible role of SESN2 in innate and adaptive immune cells, including monocytes, macrophages, natural killer (NK) cells, and T lymphocytes, has been increasingly recognized (11, 12, 17, 92). The expression and effect of SESN2 on immune cells in response to various stimuli are described in Figure 4.

### Macrophages

Macrophages are essential for the host inflammatory response, and the malfunction or apoptosis of macrophages contributes to the development of immunosuppression and a higher risk of secondary infection. A previous study revealed that DETA-NO and hypoxia induced the expression of SESN2 in primary peritoneal mouse macrophages and RAW264.7 cells in a HIF-1α-dependent manner and that SESN2 protected cells from overoxidation damage (70). Hu et al. (93) reported that oxidized low-density lipoprotein (ox-LDL) increased SESN2 expression in RAW264.7 cells, which was mediated by the JNK/c-Jun signaling pathway. SESN2 had a protective effect on ox-LDL-induced ROS production and apoptosis in RAW264.7 cells. In addition, TLR ligands (LPS, polyI:C, or peptidoglycan) upregulated SESN2 induction that was dependent on the activator protein (AP)-1 and Nrf2–ARE pathways, and SESN2 inhibited LPS-induced inflammatory insults (18). Furthermore, in a mouse myocardial infarction (MI) model, SESN2 was elevated in cardiac macrophages and had a negative regulatory effect on the pro-inflammatory response of M1 macrophages via inhibiting TNF-α, IL-6, and IL-1β expression, thereby polarizing to the M2 phenotype and alleviating excessive inflammation. Likewise, SESN2 overexpression profoundly suppressed the LPS-mediated inflammatory response of M1 and attenuated post-ischemic myocardial inflammation (94).

### Monocytes

It was reported that the human monocytic cell line THP-1 expressed SESN2 in a dose- and time-dependent manner in response to treatment with LPS, and SESN2 knockdown in THP-1 cells significantly augmented LPS-induced nuclear factor (NF)-κB phosphorylation, reduced AMPK phosphorylation, and elevated secretion of pro-inflammatory mediators [TNF-α, monocyte chemotactic protein (MCP)-1, and IL-6]. Moreover, SESN2-knockdown mice had higher plasma pro-inflammatory cytokine levels and increased monocyte recruitment to the vascular endothelium by secreting monocyte adhesion molecules [intercellular adhesion molecule (ICAM)-1 and vascular cell adhesion molecule (VCAM)-1] through inhibiting the AMPK signaling pathway and downregulating the ER stress pathway, which contributed to atherosclerosis initiation (32). Similarly, another study reported that SESN2 regulated monocyte polarization and adhesion to endothelial cells and decreased inflammatory responses by regulating the AMPK–mTOR nexus under high-glucose and dyslipidemic conditions (95). Moreover, in a sepsis mouse model and in septic shock patients, SESN2 levels were increased and peaked at 48 h in blood monocytes, and SESN2 protected organisms against inflammatory responses and septic shock, which were associated with decreased serum concentrations of IL-1β and IL-18 (12). This study highlighted the protective effect of SESN2 from sepsis and sepsis shock by maintaining mitophagy activation to inhibit NLRP3 inflammasome hyperactivation for immunological homeostasis.

### CD4^+^ T Lymphocytes

Recently, Lanna et al. (11) showed that SESN1, SESN2, and SESN3 protein expression was much higher in senescent blood-derived primary human CD4^+^ T cells than in non-senescent T cells and that downregulation of these proteins in senescent T cells showed antiaging effects, evidenced by the enhancement of cell proliferation, telomerase activity, and IL-2 synthesis. Similarly, SESN expression was much higher in 20-month-old mice than in 2-month-old mice. Notably, SESNs exhibit pro-aging functions in T cells by binding and activating MAPK pathway members, including ERK, JNK, and p38 MAPK, which are called new SESN–MAPK activation immune-inhibition complexes (sMACs). This effect is opposite to the function of antiaging activities in other cells, which implies some undiscovered mechanisms. In addition, the study revealed that more sMACs were formed in T cells from older humans (>70–85 years old) and mice (16–20 months old) than in younger humans and mice, and more importantly, the deficiency of SESNs increased splenocytes (T cells and NK cells) and restored immunity and cytokine production in mice after vaccination (11). Recently, Xiao et al. (96) found that in aortic dissection patients, SESN2 was secreted by aortic macrophages and CD4^+^ T lymphocytes but not smooth muscle cells (SMCs) and that SESN2 levels were elevated in both plasma and aortas. Moreover, upregulation of SESN2 decreased Ang II-induced SMC apoptosis and attenuated the occurrence and progression of aortic dissection. Although the protective role of SESN2 in cells has been found, further studies are also needed to investigate the possible mechanisms.

### Other Immune Cells

The NK-92 cell line has high tumoral potency and is widely used for immunotherapy in cancer (97). In an ovarian cancer xenograft mouse model, Wang et al. (92) first identified that SESN2 and SESN3 expression was much higher in intratumoral NK-92 cells than in normal human blood NK cells. Overexpression of SESN2 or SESN3 could inhibit NK-92 cell-mediated cytotoxic activation through the activation of AMPK and the suppression of mTORC1, which manifested as decreases in the natural cytotoxicity receptors (NKp44 and NKG2D) and cytotoxic factor (TNF-α) and weakened the antitumor activity of NK-92 cells, emphasizing the potential effect of SESNs on immunotherapy in cancer.

## SESN2 and Diseases

Many studies have confirmed the important impact of SESN2 on immune cells, and SESN2 levels are elevated in patients with various diseases, and their plasma levels are positively correlated with the severity of those diseases (96, 98, 99). Thus, we infer that SESN2 might be an important biomarker and immunotherapeutic target in terms of the diagnosis, treatment, and prognosis of various inflammatory-related diseases. The effects of SESN2 on different diseases are listed in Table 2.

**Table 2:** The effects of SESN2 on various inflammatory-related diseases.

**Inflammatory disorder**	**Effect**	**References**
Sepsis	Decreasing mortality rate Attenuating systemic inflammation Reducing ROS accumulation	(12, 17, 18)
Liver diseases	Protecting liver against hepatitis Inhibiting TLR-mediated pro-inflammatory response Decreasing liver injury Preventing hepatocyte death, steatohepatitis, and liver fibrosis Attenuating obesity-induced hepatic steatosis Attenuating glucose intolerance and insulin resistance	(14–18)
Ischemia–reperfusion injury	Improving post-ischemic cardiac function Decreasing heart sensitivity to ischemia insult Decreasing heart infarct areas Maintaining basal cardiac integrity Ameliorating oxidative stress insult Alleviating brain infarct areas, attenuating brain atrophy, lowering neuron apoptosis, improving the blood–brain barrier integrity, and improving long-term neurological function	(20–22, 24, 100)
Neurodegenerative diseases	Decreasing neuron apoptosis Attenuating oxidative stress Alleviating peripheral nerve injury Inhibiting inflammatory neuropathic pain	(26–28)
Cardiovascular diseases	Inhibiting chronic heart failure Attenuating smooth muscle cell apoptosis Delaying the progression of atherosclerosis	(6, 32–35, 96)
Chronic obstructive pulmonary disease	Decreasing alveolar maintenance programs Alleviating pulmonary emphysema	(36, 37, 101)
Obesity and diabetes	Maintaining glucose metabolic homeostasis Avoiding insulin resistance	(48, 102, 103)
Cancer	Inhibiting cancer cell migration Inhibiting cancer growth	(41, 43)

### Sepsis

Sepsis is defined as a life-threatening organ dysfunction caused by a dysregulated host response to infection (104). Immune dysregulation plays a vital role in the pathophysiological process of sepsis and profoundly influences its high mortality rate (105, 106). Recently, reports showed that LPS significantly upregulated the gene and protein expression of SESN2 but not SESN1 in macrophages, such as RAW264.7 cells, mouse bone marrow-derived macrophages (BMDMs), and human THP-1 macrophages. The induction of SESN2 markedly prevented cell apoptosis and liver injury from endotoxin toxicity (18). In accordance with this research, Yang et al. (17) found that LPS obviously enhanced SESN2 expression and that upregulation of SESN2 alleviated the inflammatory response by inhibiting the formation of pro-inflammatory cytokines, prevented cell death, and decreased ROS production or oxidative stress insult after LPS stimulation. Therefore, SESN2 can counteract TLR-mediated pro-inflammatory signaling and plays a protective role in LPS-induced inflammatory responses as well as cell death.

In the cecal ligation and puncture (CLP) mouse sepsis model and endotoxemia-induced sepsis model, SESN2 levels were increased, and the more severe the sepsis, the higher the expression of SESN2 and *vice versa*. Similar to the mouse model, the protein level of human SESN2 was highly upregulated in monocytes in septic shock patients, and the protein level fluctuation was considered to be associated with the serum concentrations of IL-1β and IL-18 in septic patients. Meanwhile, deficiency of SESN2 showed defective mitophagy, caspase-1 hyperactivation, and increased secretion of IL-1β and IL-18, which ultimately increased the mortality in murine sepsis (12). These data highlight the protective effect of SESN2 on maintaining inflammatory response and immunological homeostasis during sepsis; however, further investigations should be performed to explore the underlying mechanisms of SESN2 in the pathogenesis of septic complications.

### Liver Diseases

The development and progression of most liver diseases are associated with uncontrolled inflammatory responses resulting in hepatic damage. Growing evidence demonstrates that SESN2 has a marked impact on inflammation-associated pathogenesis of liver diseases (107, 108). In d-galactosamine (Gal)/LPS-induced hepatitis, TLR activation participated in SESN2 induction in liver macrophages, and SESN2 attenuated LPS-induced pro-inflammatory cytokines, hepatic cell death, and liver damage (17, 18). It was revealed that SESN2 knockdown mice exhibited an increased number of macrophages, severe hepatocyte death, and extensive liver damage (14). It is likely that SESN2 deficiency provoked high-fat diet (HFD)-mediated hepatic steatosis, hepatic inflammation, and liver fibrosis. In contrast, liver-specific SESN2 reconstitution completely reversed HFD-induced non-alcoholic steatohepatitis and liver damage. In addition, acetaminophen (APAP) caused hepatotoxicity and inflammatory response in mice, whereas administration of Ad-SESN2 ameliorated APAP-induced hepatocyte degeneration and inflammatory cell infiltration and reduced the mortality rate (13). What's more, Kim et al. (16) found that methionine/choline-deficient (MCD) diet-fed mice showed hepatic transglutaminase elevation and liver inflammation and damage, while treatment with carbon monoxide ameliorated levels of plasma AST, ALT, and inflammatory cytokines via increasing SESN2 levels. Knockdown of SESN2 impeded the protective effect of carbon monoxide on the livers of MCD-fed mice. In agreement with these observations, ablation of SESN2 exacerbated acute liver injury or obesity-induced hepatosteatosis, whereas upregulation of SESN2 exhibited a hepatoprotective effect (15). Taken together, these results suggest the pivotal role of SESN2 in the modulation of hepatic homeostasis and inflammation.

### Ischemia–Reperfusion Injury

Ischemia–reperfusion (I/R) injury is a key factor that causes energy metabolism disorders, cell damage, uncontrolled inflammation, and organ dysfunction. Moreover, the major interventions against these damages are alleviation of oxidative stress and attenuation of the inflammatory response and cell apoptosis (109, 110).

In a myocardial I/R injury model, Morrison et al. (20) found that SESN2 was significantly increased in murine cardiac tissue during ischemic conditions and that SESN2-knockout mice showed severe myocardial infarct size and post-ischemic cardiac dysfunction. Intriguingly, SESN2 functioned as an ischemic-scaffold protein in the AMPK–LKB1 axis, promoting AMPK activation to reduce ischemic injury. In line with this study, Quan et al. (21) determined that the dynamic decline of SESN2 paralleled the increasing sensitivity to ischemic insults in aged hearts. Aged and SESN2-knockout hearts exacerbated I/R-induced cardiac damage, as evidenced by worsened cardiac dysfunction and increased MI size *in vivo* and *in vitro*. Rescue of SESN2 significantly improved cardiac function and decreased infarction size in aged hearts subjected to I/R insults. The mechanisms underlying SESN2 in downregulating the sensitivity of aged hearts to ischemic stress were mainly dependent on the interaction with the AMPK signaling pathway to modulate PGC-1α, which could affect mitochondrial biogenesis and attenuate the apoptosis of cardiocytes following ischemic insults (22).

In an acute hypoxia rat model of stroke by permanent middle cerebral artery occlusion (MCAO), Hi95 was highly increased in the rat brain and showed a protective role against ischemia brain injury (3). Likewise, in a transient global I/R rat model, SESN2 was obviously augmented in the hippocampal CA1 subfield, and silencing of SESN2 exacerbated the extent of oxidative stress damage, neuronal injury, and hippocampal neuron apoptosis (23). Consistent with these results, further studies confirmed that SESN2 played a protective role against cerebral I/R injury by alleviating brain infarct areas, attenuating brain atrophy, reducing neuron apoptosis, improving the blood–brain barrier integrity, and improving long-term neurological function (24, 71, 100). Additionally, SESN2 was increased in cortical renal tubules during I/R-induced acute kidney injury, and p53-SESN2 prevented the I/R damage-induced apoptosis of renal cells by activating autophagy and mitophagy (25).

### Neurodegenerative Diseases

It has been demonstrated that SESN2 contributes to chronic inflammation associated with neurodegenerative diseases, such as Alzheimer's disease (AD) and Parkinson's disease (PD). SESN2 was upregulated in primary rat cortical neurons treated with amyloid-β/Aβ (a hallmark of AD-caused cytotoxicity and neuronal death) both *in vitro* and *in vivo*, and that elimination of SESN2 exacerbated Aβ-induced neurotoxicity, which indicated that SESN2 upregulation by Aβ positively modulated autophagy to protect against neurotoxicity (26). A clinical study showed that the expression of SESN2 was altered in brains with human immunodeficiency virus (HIV)-associated neurocognitive disorders (HAND). SESN2 was mainly located in neuronal soma in cases of HAND, whereas it colocalized with p-Tau in neurofibrillary lesions in AD brains, showing a protective role against neuronal oxidative stress (27). Another clinical study documented that SESN2 levels were more highly increased in the serum of AD patients whose disease course was <2 years when compared to patients with mild cognitive impairment or elderly patients older than 60 years of age, suggesting a potential application as an early protein marker for the detection of AD (28). In accordance with the findings obtained from the model of AD, adoption of a PD model by Hou et al. (84) indicated that SESN2 enhanced autophagy activity and inhibited cell death in a PD model *in vitro*. Additionally, it has been reported that SESN2 expression is increased in both patients with PD and a 1-methyl-4-phenylpyridinium (MPP+)-induced PD cell model and that SESN2 plays a protective role against MPP+-induced neurotoxicity by regulating mitochondrial function, oxidative stress, and cell apoptosis (111). These results indicate that SESN2 has a protective effect on neurodegenerative diseases.

### Cardiovascular Diseases

Cardiovascular diseases belong to age-related pathologies, and inflammatory conditions are one of the characteristics of these diseases. Studies have shown that SESN2 plays a pivotal role in the occurrence and development of various cardiac pathophysiologies. As reported, SESN2 levels were elevated in patients with chronic heart failure, coronary artery diseases, aortic dissection, and atrial fibrillation (96, 99, 112, 113). Lee et al. (6) originally described the regulatory function of SESNs in heart function. They further demonstrated that loss of dSESN was associated with age-associated pathologies, such as cardiac malfunction, muscle degeneration, and triglyceride accumulation. One mechanism by which SESN2 affected cardiac function is related to its beneficial effect against cardiomyocyte hypertrophy. As reported, in a phenylephrine-induced hypertrophy model, SESN2 expression was decreased in the process of cardiac hypertrophy, and SESN2 retarded the cardiomyocyte hypertrophy process by inhibiting ERK1/2 signaling (33). And another mechanism of SESN2 protecting against cardiac remodeling is via enhancing its antioxidant effect on the Nrf2–Keap1 pathway (34). What's more, in human umbilical vein endothelial cells, treatment with angiotensin (Ang) II increased SESN2 expression, and knockdown of SESN2 exacerbated Ang II-induced toxicity (35). Similarly, SESN2 attenuated Ang II-induced apoptosis of SMCs via regulating the Nrf2 pathway and decreased the occurrence of aortic dissection (96).

Moreover, in an atherosclerosis model, SESN2 protected blood vessels by decreasing the formation of atherosclerotic plaques or other hallmarks in endothelial cells (32). Chronic inflammation precedes atherosclerosis progression, and SESN2 prevented the development of atherosclerosis by activating the AMPK pathway and then by reducing inflammation response. Taken together, SESN2 might be a beneficial target for amelioration of cardiovascular diseases, indicating its potential therapeutic role in cardiovascular diseases.

### Chronic Obstructive Pulmonary Disease

Chronic obstructive pulmonary disease (COPD) is characterized by chronic inflammatory and immune responses mainly from the small airways to lung parenchyma, and aberrant immunity and oxidant/antioxidant imbalance might be the central pathway in the pathogenesis of COPD (114, 115).

In the mouse model of COPD, Wempe et al. (36) found that inactivation of SESN2 protein decreased airways and elastic fiber fragmentation of alveolar walls, rescued pulmonary emphysema, and improved pulmonary function via selectively activating transforming growth factor-β signaling. Similarly, SESN2 was significantly upregulated in emphysematous lungs of patients with advanced COPD, and SESN2-knockout mice increased alveolar maintenance and reduced cigarette smoke-induced pulmonary emphysema by enhancing the expression of platelet-derived growth factor receptor β (37). Mechanistically, SESN2 inhibited platelet-derived growth factor receptor β-mediated lung regeneration and injury repair by promoting autophagic degradation of Keap1 to activate the Nrf2–Keap1 pathway that protected against pulmonary emphysema (101, 116). Therefore, SESN2 serves as a negative regulator and a biomarker in the development of COPD, and COPD patients might benefit from SESN2 antagonists in clinical management.

### Metabolic-Related Diseases

#### Obesity and Diabetes

Metabolic disorders such as diabetes and obesity are associated with mTORC1 activation (117), and inflammation seems to be involved in the biological process of these diseases (118). Obesity can cause glucose intolerance and insulin resistance. Recently, it was found that SESN2 was upregulated upon hypernutrition and obesity in mouse liver, which was critical for maintaining glucose metabolic homeostasis in hepatocytes, and that deficiency of SESN2 exacerbated obesity-induced hepatosteatosis. Knockout of SESN2 exacerbated obesity-induced insulin resistance and diabetic progression, which were associated with impaired glucose homeostasis and reduced insulin sensitivity (48). Similarly, Chai et al. (102) reported that insulin obviously increased SESN2 expression in mouse primary hepatic cells and hepatic tumor cell lines, which was related to attenuation of proteasome-mediated SESN2 degradation. Thus, SESN2 showed a negative feedback effect on insulin signaling transduction (103).

#### Cancer

Inflammation is a hallmark in the development and progression of cancer, and cancer-related inflammation fosters the initiation and growth of cancers (119). Recent studies have indicated that a low level of SESN2 is associated with progression and poor prognosis in different types of cancer patients or tumor models. It was reported that SESN2 acted as a potential suppressor in the colon during intestinal inflammation and colon cancer progression and contributed to maintaining intestinal homeostasis. SESN2-deficient mice had accelerated colonic epithelial cell apoptosis and inflammation during colitis. Moreover, SESN2 was strongly decreased in colon adenocarcinoma tissues, and its expression was downregulated in correlation with the progression of colon cancer. Loss of SESN2 promoted colon cancer growth and reduced susceptibility to chemotherapeutic treatments, as evidenced by dramatically increased tumor size and burden (40). In addition, Seo et al. (41) reported that SESN2 played a beneficial role in inhibiting colorectal cancer cell migration *in vitro*.

More recently, Liang et al. (42) found that SESN2 was significantly decreased in human bladder cancer cells, that isorhapontigenin could upregulate SESN2 expression in a MAPK8-JUN-dependent manner, and that SESN2 induction could inhibit human bladder cancer growth by activating autophagy. Additionally, SESN2 expression was lower in hepatocellular carcinoma tissues than in non-cancerous tissues, and low SESN2 levels were related to positive lymph node metastasis, advanced tumors, and poor prognosis in hepatocellular carcinoma patients (43). Mechanistically, SESN2 inhibited hepatocyte proliferation and carcinoma development by activating the Nrf2 pathway and suppressing the mTOR pathway (44). Collectively, SESN2 has anticancer effects, and modulation of SESN2 expression might contribute to the treatment and prognosis of cancers.

#### Aging

Accumulation of ROS and activation of mTORC1 are general features of aging and age-associated disorders. As SESN2 has antioxidant property and mTORC1 suppression ability, it seems plausible that SESN2 could modulate aging and age-related diseases. It has been reported that a p53-overexpressing mice model accompanied with the elevated expression of SESN1 and SESN2 could delay aging-associated damage and increase longevity (120). Additionally, dSESN inactivation resulted in the reduction of health span and acceleration of age-related tissue degeneration including muscle degeneration, cardiac dysfunction, and lipid accumulation by preventing excessive ROS accumulation and TORC1 activation (6). Moreover, in *Caenorhabditis elegans*, SESN1, the only SESN ortholog, acted as a positive modulator of health span, and a mutant of SESN1 showed muscle dysregulation (47). Given all of the above, we confer that SESN2 might provide a key feedback modulation of aging-associated pathologies. And it will be interesting to ascertain the contribution of SESN2 to these antiaging functions in further investigations.

## Conclusions and Perspectives

SESN2 is regarded as a stress-induced protein, and substantial evidence has demonstrated its vital effect in regulating diverse cellular functions, including cell fate, energy metabolism, inflammation, liver diseases, and carcinogenesis. Over the past two decades, tremendous progress has been made toward understanding the biological structure and functions of SESN2 in physiopathology, but certain limitations remain. Firstly, SESN family members share high sequence homology, which might have redundant functions. However, there is a lack of studies on SESN1 and SESN3, and it will be important for future studies to identify similarities and differences between the biochemical functions of the SESN family under different stress conditions. Additionally, the potential role of SESN2 in classical inflammatory diseases, including sepsis, is still uncertain; thus, the biochemical and biological basis of SESN2 requires a better understanding, and the precise molecular mechanism by which SESN2 regulates the inflammatory process remains to be investigated. More importantly, as most studies are based on cell culture or animal models, to what extent these findings are applicable to humans is still unclear. Further studies on the upstream and downstream regulators of SESN2 may contribute to a deep understanding of the biological, physiological, and pathophysiological effects of SESN2 in various inflammatory disorders. It will be necessary to use SESN2 transgenic or SESN2-deficient animal models and carry out more clinical trials to clarify the mechanisms by which SESN2 elicits protective responses and may serve as a drug target in the prevention and treatment of inflammatory diseases.

## Author Contributions

L-XW drafted the manuscript. X-MZ revised the manuscript. Y-MY contributed to the conception of the review and helped perform the constructive discussions.

### Conflict of Interest

The authors declare that the research was conducted in the absence of any commercial or financial relationships that could be construed as a potential conflict of interest.

## Abbreviations

mTOR: mammalian target of rapamycin
ROS: reactive oxygen species
FoxO: forkhead box transcription factor belonging to the O-subclass
AMPK: AMP-activated protein kinase
TSC: tuberous sclerosis complex protein
Nrf2: NF-E2-related factor 2
AP-1: activator protein 1
Ucp1: uncoupling protein 1
Keap1: Kelch-like ECH-associated protein 1
VEGF: vascular endothelial growth factor
PI3K/Akt: phosphoinositide-3 kinase/Akt
PAMP: pathogen-associated molecular patterns
ULK1: Unc-51-like protein kinase 1
UPR: unfolded protein response
PERK: PER-like endoplasmic reticulum kinase
eIF2α: eukaryotic translation initiation factor 2α
IRE1: inositol-requiring enzyme 1
XBP1: X-box binding protein 1
ATF6: activating transcription factor 6
ATF4: activating transcription factor 4
JNK: c-Jun N-terminal kinase
sMAC: sestrin-MAPK activation immune-inhibition complex
NLRP3, NLR family: pyrin domain containing 3
I/R: ischemia–reperfusion
PGC-1α: peroxisome proliferator-activated receptor gamma coactivator-1
MCAO: permanent middle cerebral artery occlusion
HAND: human immunodeficiency virus (HIV)-associated neurocognitive disorders
COPD: chronic obstructive pulmonary disease.
